# Inhibition of Gastric Lipase as a Mechanism for Body Weight and Plasma Lipids Reduction in Zucker Rats Fed a Rosemary Extract Rich in Carnosic Acid

**DOI:** 10.1371/journal.pone.0039773

**Published:** 2012-06-22

**Authors:** María Romo Vaquero, María-Josefa Yáñez-Gascón, Rocío García Villalba, Mar Larrosa, Emilie Fromentin, Alvin Ibarra, Marc Roller, Francisco Tomás-Barberán, Juan Carlos Espín de Gea, María-Teresa García-Conesa

**Affiliations:** 1 Research Group on Quality, Safety and Bioactivity of Plant Foods, Dept. Food Science and Technology, Centro de Edafología y Biología Aplicada del Segura-Consejo Superior de Investigaciones Científicas, Campus de Espinardo, Murcia, Spain; 2 Naturex SL, Valencia, Spain; 3 Naturex Inc., South Hackensack, New Jersey, United States of America; 4 Naturex SA, Site d'AgroParc, Avignon, France; Sapienza University of Rome, Italy

## Abstract

**Background:**

Rosemary (*Rosmarinus officinalis* L.) extracts (REs) exhibit hepatoprotective, anti-obesity and anti-inflammatory properties and are widely used in the food industry. REs are rich in carnosic acid (CA) and carnosol which may be responsible for some of the biological activities of REs. The aim of this study was to investigate whether inhibition of lipase activity in the gut may be a mechanism by which a RE enriched in CA (40%) modulates body weight and lipids levels in a rat model of metabolic disorders and obesity.

**Methods and Principal Findings:**

RE was administered for 64 days to lean (*fa*/+) and obese (*fa*/*fa*) female Zucker rats and body weight, food intake, feces weight and blood biochemical parameters were monitored throughout the study. Lipase activity (hydrolysis of *p*-nitrophenylbutyrate) was measured in the gastrointestinal tract at the end of the study and the contents of CA, carnosol and methyl carnosate were also determined. Sub-chronic administration of RE moderately reduced body weight gain in both lean and obese animals but did not affect food intake. Serum triglycerides, cholesterol and insulin levels were also markedly decreased in the lean animals supplemented with RE. Importantly, lipase activity was significantly inhibited in the stomach of the RE-supplemented animals where the highest content of intact CA and carnosol was detected.

**Conclusions:**

Our results confirm that long-term administration of RE enriched in CA moderates weight gain and improves the plasma lipids profile, primarily in the lean animals. Our data also suggest that these effects may be caused, at least in part, by a significant inhibition of gastric lipase and subsequent reduction in fat absorption.

## Introduction

Obesity is a global health issue resulting from a combination of high-energy diets, sedentary lifestyles and genetic make-up and is accompanied by a cluster of chronic metabolic diseases which include cardiovascular diseases (CVDs), hypertension, type-2 diabetes and non-alcoholic steatohepatitis. The incidence and impact of these major health threats have risen to alarming proportions in Western societies and there is a great need for therapeutic and preventive measures [1]. The pharmaceutical industry has invested efforts and resources in producing anti-obesity drugs such as lipid digestion inhibitors; however, only an inhibitor obtained from actinobacterium, orlistat, is currently approved and authorized in Europe for obesity treatment [2].

Obesity and metabolic disorders are characterized by an excessive weight due to enlarged fat deposition and by an associated chronic inflammation. Since current medical treatments often fail to stop the progress of these diseases, polyphenol-rich foods or derived by-products with anti-inflammatory properties are being widely investigated as a potential additional strategy to combat obesity [1]. Numerous published studies have evaluated the anti-obesity properties of polyphenols. Like this, an orange juice containing anthocyanins, flavanones and cinnamic acids has been shown to reduce body weight and fat accumulation in a mouse model of obesity [3]. More recently, it has been reported that an aqueous roiboos extract rich in polyphenols reduced the levels of cholesterol, TGs and fatty acids as well as liver steatosis in hyperlipidemic mice [4]. The mechanisms by which polyphenols may exert these anti-obesity effects are only beginning to emerge and may involve a combination of multiple molecular effects such as modulation of metabolic-, antioxidant-, and anti-inflammatory-gene and protein expression [1].

*Rosmarinus officinalis* L (Lamiaceae) is a plant widely distributed in Europe, Asia and Africa much utilized in the food industry for its functional properties as well as for its beneficial health properties. The biological activities of the plant have been attributed to two groups of compounds: the volatile fraction and the phenolic compounds. This latter group mainly contains a flavonoid fraction, rosmarinic acid, and some diterpenoid compounds structurally derived from CA, e.g. carnosol, rosmanol, etc. The most abundant compounds, CA and rosmarinic acid, exhibit a wide range of activities, including antioxidant, anti-inflammatory and hepatoprotective effects [5]. Currently, considerable scientific interest is directed toward the effects of REs on obesity and obesity-related diseases. Rosemary leaf extracts and CA have been shown to reduce body weight, fat mass gain and serum lipids levels in male mice fed a high-fat diet [6], [7] and in a leptin-deficient (*ob/ob*) male mouse model [8]. Since REs, CA and carnosol have been shown to inhibit porcine pancreatic lipase in vitro [6], [7], [9], the reported anti-obesity effects of rosemary have been attributed to the inhibition of this activity [6], [8].

To gain further insight in the mechanisms by which REs and (or) CA may exert their anti-obesity properties, we have determined the lipase activity in the gut, liver and pancreas of obese (*fa*/*fa*) leptin receptor-deficient female Zucker rats and their lean (*fa*/+) counterparts following long-term administration of a RE highly enriched in CA (40%). The effects of the RE on body weight, food intake, biochemical blood parameters and liver lipids were analyzed and compared between the obese and the lean animals. In addition, we have determined and quantified the presence and content of CA, carnosol and methyl carnosate in these organs.

## Materials and Methods

### Materials

The RE used in this study (reference GAX00011 batch number A273/045/A10) was kindly provided by Naturex (Valencia, Spain) and was prepared according to a patented method [10] as follows: rosemary leaves were extracted with 96% ethanol at room temperature and the ethanolic extract was filtered and concentrated under reduced pressure at 45°C to make a concentrated native extract. The native extract was then diluted with aqueous sodium bicarbonate (NaHCO_3_) at pH 8.5–9 to dissolve CA and other organic acids, while other substances insoluble in alkaline conditions were precipitated out. The solution was filtered and the filtrate further concentrated under reduced pressure. The concentrated solution was then acidified with phosphoric acid (H_3_PO_4_) to pH 4.9 to precipitate the acid insoluble substances including CA, carnosol and CA derivatives. The solid precipitate was subsequently washed with water and filtered to remove impurities. The purified solid product was finally dried in a vacuum oven and milled into powder. CA and carnosol standards were purchased from Sigma-Aldrich S.A. (Madrid, Spain). Methanol (MeOH), acetone and acetonitrile (ACN) were obtained from Merck (Darmstadt, Germany). Milli-Q system (Millipore, USA) ultrapure water was used throughout the study. All other chemicals were of analytical grade.

### Animals and Experimental Design

Fourteen female Zucker lean (*fa*/+) (Le) and 10 obese (*fa*/*fa*) (Ob) rats aged 5 weeks (105.5±13.3 g and 148.5±22.9 g, respectively) were purchased from Harlan Laboratories Models S.L. (Barcelona, Spain). This study was carried out at the Experimental Animal Facility of the University of Murcia (Spain; Registration number: REG A ES 300305440012) in strict accordance with the recommendations of the European Union regarding animal experimentation (Directive of the European Council 86/609/EC). The protocol was approved by the local animal ethics committee and the local government (Murcia Autonomous Government of Agriculture, Fisheries and Livestock, *Permit Number: C1311031102*). Rats were housed 2–4 to a cage in a room with controlled temperature (22±2°C), 55%±10% relative humidity, and a 12 h light-dark cycle. They were fed with a standard chow (composition: 14.3% protein, 4.0% fat, 48.0% carbohydrate, 4.1% crude fiber, 18.0% neutral detergent fiber and 4.7% ash; energy density, 2.9 kcal/g) for several days before the start of the experiments. Animals were randomly assigned to two experimental groups: i) control groups (CTLe, n = 7 and CTOb, n = 5) fed with the standard chow alone and ii) treated groups (RELe n = 7 and REOb, n = 5) fed with the standard chow supplemented with RE (0.5% w/w). Diet and tap water were administered *ad libitum*. Dietary intervention lasted for 64 days. Body weight, feces weight and food and water intake were recorded throughout the study. At the end of the experimental period, non-fasting animals were anaesthetized by intramuscular injection with a mixture (1∶1, v/v; 1 mL/kg of body weight) of ketamine (Imalgene 1000; Merial Laboratories, Barcelona, Spain) and xylazine (Xilagesic 2%; Calier Laboratories, Barcelona, Spain) and sacrificed by heart puncture exsanguination. All efforts were made to minimize suffering. Liver, pancreas as well as the contents of stomach, duodenum (first 10 cm of the small intestine), and small intestine (jejunum+ileum) were rapidly taken, weighed, frozen in liquid nitrogen and stored at −80°C until further analysis.

### Sample preparation for the analysis of phenolic compounds

Fresh solutions of RE were prepared by dissolving 0.1 g of the powder in 2.0 mL of ACN (1 h, 40°C). The solution was further diluted (1/50) with ACN and filtered (0.45 µm) prior to injection (20 µL) into the HPLC-DAD-MS/MS system. Pancreas and liver samples (∼0.5 g) were vigorously homogenized with 2.5 mL of MeOH/water/HCl (79.9∶20∶0.1) using an IKA T10 Ultra-Turrax (Janke & Kunkel, Ika-Labortechnick, Germany) at 24000 rpm (4°C) for 1 min and then exposed to ultrasound for 10 min. The mixture was centrifuged (Eppendorf 5804R, Eppendorf AG, Hamburg, Germany) at 2880 *g* (4°C) for 5 min. The methanolic phase was evaporated in a SpeedVac concentrator (Savant SPD121P, Thermo Scientific, Alcobendas, Spain) equipped with a refrigerated vapor trap RVT4104 (Thermo Scientific, Alcobendas, Spain) and a vacuum pump v-710 (BÜCHI Labortechnik AG, Postfach, Switzerland), then diluted with 200 µL of MeOH and filtered (0.45 µm) prior to injection (20 µL) to the HPLC-DAD-MS/MS system. Gut contents (50–500 mg) were also homogenized with cold MeOH/water/HCl (50∶49.9∶0.1) (0.3–1.5 mL) using an IKA T10 Ultra-Turrax (Janke and Kunkel, Ika-Labortechnick, Germany) at 24000 rpm at 4°C for 1 min and then exposed to ultrasound for 10 min. The mixture was then centrifuged at 2880 *g* at 4°C for 5 min and the supernatant kept at 4°C. Each pellet was extracted twice with the same methanolic solution. Both supernatants were pooled, filtered (0.45 µm) and injected (20 µL) into the HPLC-DAD-MS-MS system.

### HPLC-DAD-MS/MS analyses

Analyses of the phenolic compounds present in the RE and in the different organs and gastrointestinal contents were performed on an Agilent 1100 HPLC system equipped with a photodiode-array detector and an ion-trap mass spectrometer detector in series (Agilent Technologies, Waldbronn, Germany). Chromatographic separation was carried out on a reversed phase Mediterranean C18 column (4.6×250 mm, 5 µm particle size) (Tecknokroma, Barcelona, Spain), operating at room temperature and using water/formic acid (99∶1, v/v) (phase A) and ACN (phase B) at a flow rate of 0.5 mL/min. The gradient was as follows: 0–10 min, 10%–70% B; 10–45 min, 70%–90% B; 45–46 min 90–98% B; 46–50 min; 98–10% B. Finally, the column was re-equilibrated for 5 min. The ion-trap mass spectrometer was equipped with an electrospray ionization system (ESI, capillary voltage, 4 kV). Nitrogen was used as nebulizer gas at a pressure of 65 psi and as dry gas at a flow rate and temperature of 9 L/min and 350°C, respectively. Mass scan (MS) and MS/MS daughter spectra were operated in negative ionization mode with a mass scan range from *m/z* 100 to 800. Collision-induced fragmentation experiments were performed in the ion trap using helium as collision gas with collision energy of 1V. UV chromatograms were recorded at 285, 330 and 360 nm. The different compounds and metabolites were identified according to their UV spectra, molecular weight and MS-MS fragments. In addition, CA and carnosol were quantified in the rosemary extracts and in the tissue samples in comparison with authentic standards using UV signal at 285 nm.

**Biochemical and hematological parameters:** Blood samples were taken by tail incision [11] at 7, 21 and 35 days after the initiation of the study and by cardiac puncture, at the end of the study, in heparinized tubes. Hematological parameters were directly measured in the un-coagulated blood using an automatic analyzer (Abacus Junior Vet, CVM S.L., Navarra, Spain) with specific software to analyze rat blood parameters. The remaining blood was centrifuged at 3000 *g* at room temperature for 15 min in a Sigma 1–13 microcentrifuge (Braun Biotech. International, Germany) to obtain plasma. Plasma samples were immediately frozen at −80°C prior to analysis of biochemical parameters. Levels of total glucose, total cholesterol, HDL-cholesterol (HDL-c), LDL-cholesterol (LDL-c), triglycerides (TGs), and the hepatic leakage enzymes: alkaline phosphatase (ALP), aspartate aminotransferase (AST), and alanine aminotransferase (ALT) were measured using an Olympus AU600 autoanalyzer (Olympus Diagnostica, Hamburg, Germany). Insulin concentration was quantified using an ultrasensitive rat insulin enzyme immunoassay kit (Mercodia AB, Uppsala, Sweden) in a microplate reader (TECAM infinite M200, Tecan Austria GmbH 5082, Grodig Austria) with a 450 nm filter [12].

**Hepatological examination:** Sections of snap frozen liver from each animal were embedded on the OCT cryostat embedding compound (Tissue-Tek, Torrance, CA) and sectioned (10 µm) with a cryostat (CM 3050S, Leica Microsystems GmbH). Sections were stained using the oil red O method [13] and observed under a light microscope (Nikon Eclipse 90i) at 20× magnification to detect neutral lipids. Images were captured using a digital camera (Nikon Dxm 1200c) and the percentage of area occupied by oil red O stained lipid droplets was calculated using the Leica Qwin imaging software. Five fields per section were analyzed.

Total TGs and cholesterol were analyzed in the liver following a previously reported method [14]. Liver fragments (∼100 mg) were homogenized with 1 mL of RIPA buffer (50 mM Tris-HCl, pH 7.5, 150 mM NaCl, 1 mM EDTA, 0.1% SDS, 0.5% deoxycolic acid, 1% Triton X-100). Tissue extract (100 µL) was then mixed with 1.6 mL of CHCl3:MeOH (2∶1) for 16 h at 4°C followed by addition of 200 µL of 0.6% NaCl and the solution was centrifuged at 2.000 *g* for 20 min. The organic layer was removed and dried using a SpeedVac concentrator. The resulting pellet was dissolved in 100 µL phosphate buffered saline (PBS) containing 1% Triton X-100. TGs and cholesterol content were measured using an Olympus AU600 autoanalyzer (Olympus Diagnostica, Hamburg, Germany).

### Enzymatic activity

On the day of use, the frozen organ or gut content samples were quickly weighed (50–250 mg) and homogenized in ice-cold Tris-HCl buffer (0.1 M) pH 8.0 (400–500 µL) using an Ultra Turrax IKA-T10 basic tissue blender (Janke and Kunkel, Ika-Labortechnick, Germany). Homogenates were clarified by centrifugation at 20817 *g* for 10 min at 4°C and supernatants were collected for the analysis of protein and enzyme activity. The total protein content was measured by the *DC* colorimetric assay at 750 nm (BioRad, Barcelona, Spain) based on a bovine serum albumin standard curve. Lipase activity was tested by monitoring the cleavage of the substrate *p*-nitrophenylbutyrate (≥98%, Sigma-Aldrich S.A., Madrid, Spain) (PNPB) to release *p*-nitrophenol [15]. A stock solution of PNPB (2.0 mM) was prepared in DMSO. The reaction mixture (final volume, 1100 µL) contained the tissue supernatant diluted to a suitable protein concentration in Tris-HCL buffer (0.1 M) pH 8.0 and PNPB (25 µL, 2.0 mM) which was added to start the reaction. After a brief mixing and autozero, the absorbance at 400 nm was recorded for 5 min at 37°C using a UV/visible spectrophotometer (Jasco V-630, Tokyo, Japan). Background hydrolysis was measured by monitoring the autohydrolysis of the substrate (no enzyme added) under the conditions of the assay. The initial rate of absorbance increase minus the background value was used as the measure of enzymatic activity. The absorbance was converted to concentration of *p*-nitrophenol using a standard curve. Activity values were expressed in nanokatals (nkat)/g protein (1 U = 16.67 nkats) and are shown as the mean value ± SD of at least triplicate independent measurements.

Inhibition of pancreatic lipase (lipase from porcine pancreas, Type II, 100–400 units/mg protein, Sigma-Aldrich S.A., Madrid, Spain) by CA and carnosol was also determined by the method described above. CA or carnosol (5, 10 and 20 µg) were mixed with 20 µL of pancreatic lipase (1 mg/mL) in 0.1 M Tris-HCl buffer (pH 8) and 25 µL PNPB (2 mM) were added to start the reaction (final volume, 1100 µL). Following incubation at 37°C for 5 min, absorbance was read at 400 nm.

### Statistical analysis

Results are expressed as the mean value ± SD. Differences between groups were compared using an unpaired Student's *t* test. Results with a two-sided *P*-value <0.05 were considered statistically significant. *P*-values <0.1 are also indicated.

## Results

### Characterization of the RE phenolic acid composition using HPLC-DAD-MS/MS

**Figure 1:**
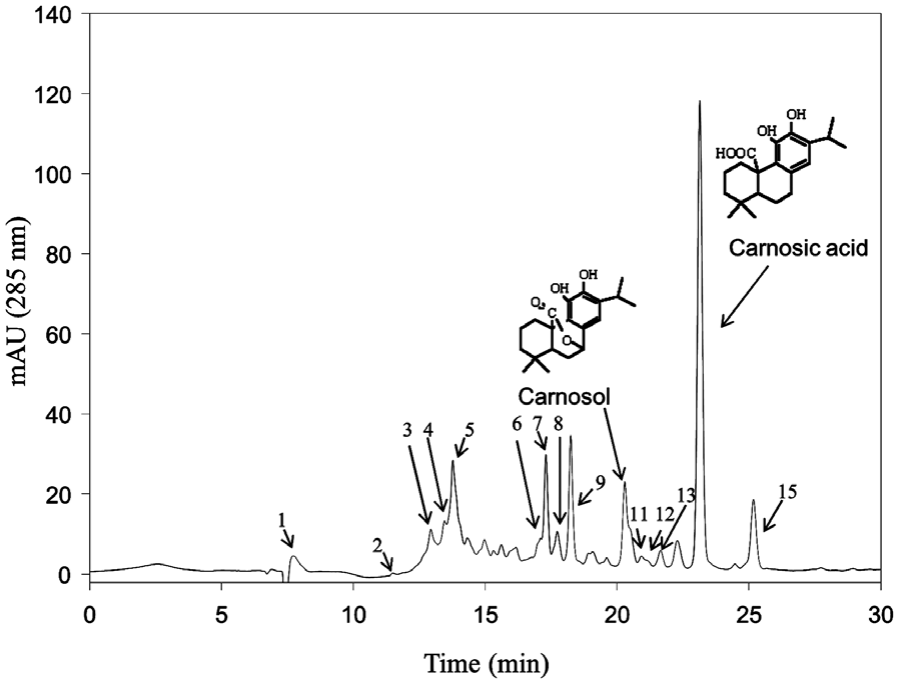
Main phenolic compounds present in the rosemary extract (RE). Chromatogram (285 nm) corresponding to the HPLC-DAD-MS analysis of the RE extract. The structure and peak identification of carnosol (peak 10) and CA (carnosic acid, peak 14) are indicated. Peak numbers correspond to each of the phenolic compounds identified in the extract and listed in Table 1.

HPLC-DAD-MS/MS analysis of the RE extract revealed that a total of 15 phenolic compounds were present in the extract distributed in three major categories: phenolic acids, diterpenes and flavonoids. Figure 1 shows the chromatographic profile (285 nm) of the RE. The compounds were characterized by their retention times, UV-Vis and mass spectrum, and identified by comparison with published data or commercial standards. A complete list with all the compounds identified in the RE is shown in Table 1. Our data confirmed that the most abundant compound was CA (38.9±1.7%). We were also able to quantify the content of carnosol (6.5±0.1%) and methyl carnosate (6.9±0.6%). The RE was mixed with the standard feed (0.5%, w/w) and its stability was tested by monitoring the content of CA using HPLC-DAD-MS/MS. The levels of CA in the chow remained stable throughout the experimental period (90.0±16.5% of the initial quantity added).

**Table 1:** List of phenolic compounds identified in the rosemary extract (RE) by their retention times, UV-Vis and mass spectra and by the use of standards.

Peak Number	Compound	Retention time (min)	Absorbance maxima (nm)	[M–H]^−^	Major fragments detected (m/z)	g/100 g
1	Caffeic acid hexoside	8.11	-	341	179, 161	-
2	Medioresinol	11.88	-	387	369, 207, 163	-
3	Isorhamnetin 3-O-hexoside	13.19	275, 340	477	462, 315, 300	-
4	Homoplantaginin	13.59	275, 334	461	446, 372, 299	-
5	Rosmarinic acid	13.88	285sh*, 332	359	223, 197, 179, 161	-
6	Rosmanol	17.21	289	345	301, 283	-
7	Cirsimaritin	17.39	274, 334	313	298, 283	-
8	Epirosmanol	17.68	288	345	329, 301, 283	-
9	Epiisorosmanol	18.26	-	345	301	-
10	Carnosol	20.22	284	329	285	6.5±0.1
11	Rosmadial	20.83	290	343	315, 299, 287	-
12	Epiisorosmanol ethyl ether	21.11	288	373	329, 283	-
13	4′-Methoxytectochrysin	21.67	-	297	269, 282	-
14	Carnosic Acid	22.88	284	331	287	38.9±1.7
15	Methyl carnosate	24.89	282	345	331, 301, 286	6.9±0.6

* : shoulder

### Subchronic consumption of RE rich in CA was not toxic to the animals

**Figure 2:**
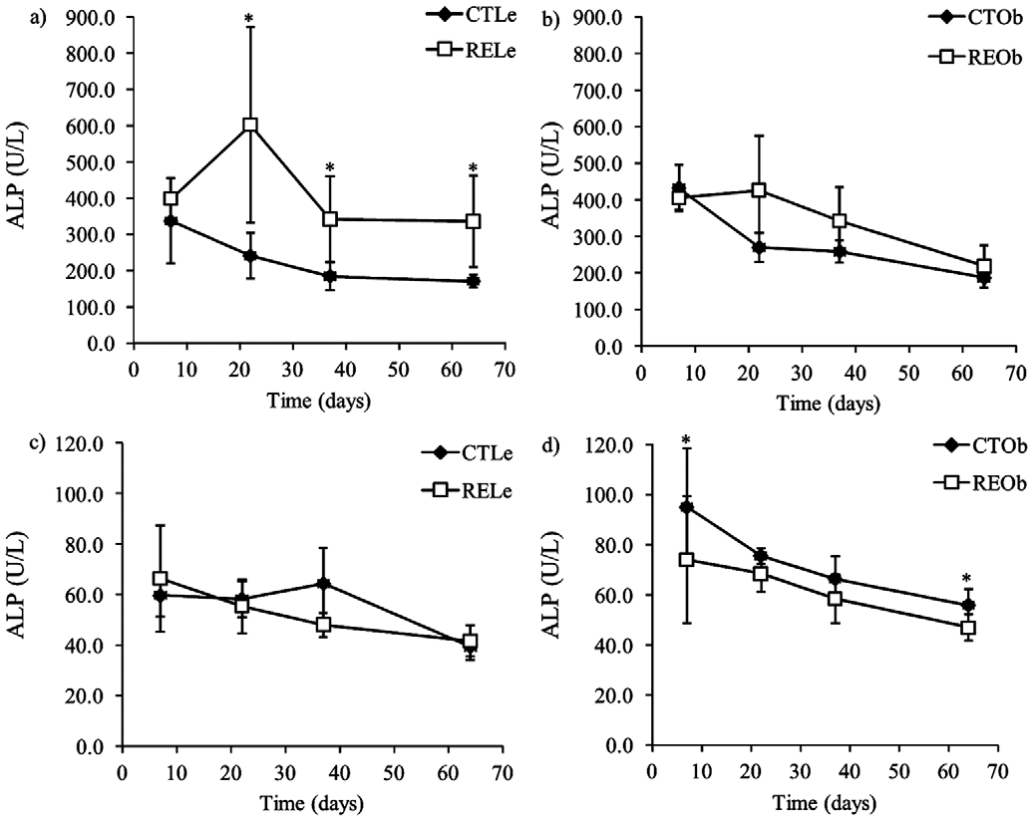
Effects of the consumption of RE on plasma levels of the hepatic enzymes ALP and ALT (U/L) in Zucker female rats. Lean (Le) and obese (Ob) rats fed the control diet (CT) or the diet supplemented with RE (RE). Data are presented as the mean value ± SD (n = 7 for lean animals and n = 5 for obese animals). * *P*<0.05 compared to their respective CT values.

All animals survived the experimental procedure and no abnormal signs or behavioral changes were observed throughout the study. The results of the analysis of the hematological parameters are shown in Table S1. Values were all within the normal range and indicate that, in general, sustained consumption of the RE enriched in CA was not toxic to the animals. We observed a small but significant increase in the lymphocyte counts in both lean and obese animals consuming the RE as well as an increase in the mid-cell fraction in the obese animals supplemented with RE. We also determined plasma levels of liver damage markers in all the animals (Table S2 and Figures 2a to 2d). The levels of AST were similar in the lean and the obese animals and were not affected by the consumption of RE. The levels of ALP, which were also similar in the lean and the obese animals, were slightly increased after supplementation with the RE, significantly in the lean animals (Figures 2a and 2b). ALT was slightly higher in the obese animals than in the lean ones and supplementation with RE caused a very small decrease in the plasma levels of this enzyme, which was shown to be significant in the obese animals (Figures 2c and 2d).

### RE rich in CA reduced body weight and increases fecal weight without affecting food intake in lean and obese Zucker rats

**Figure 3:**
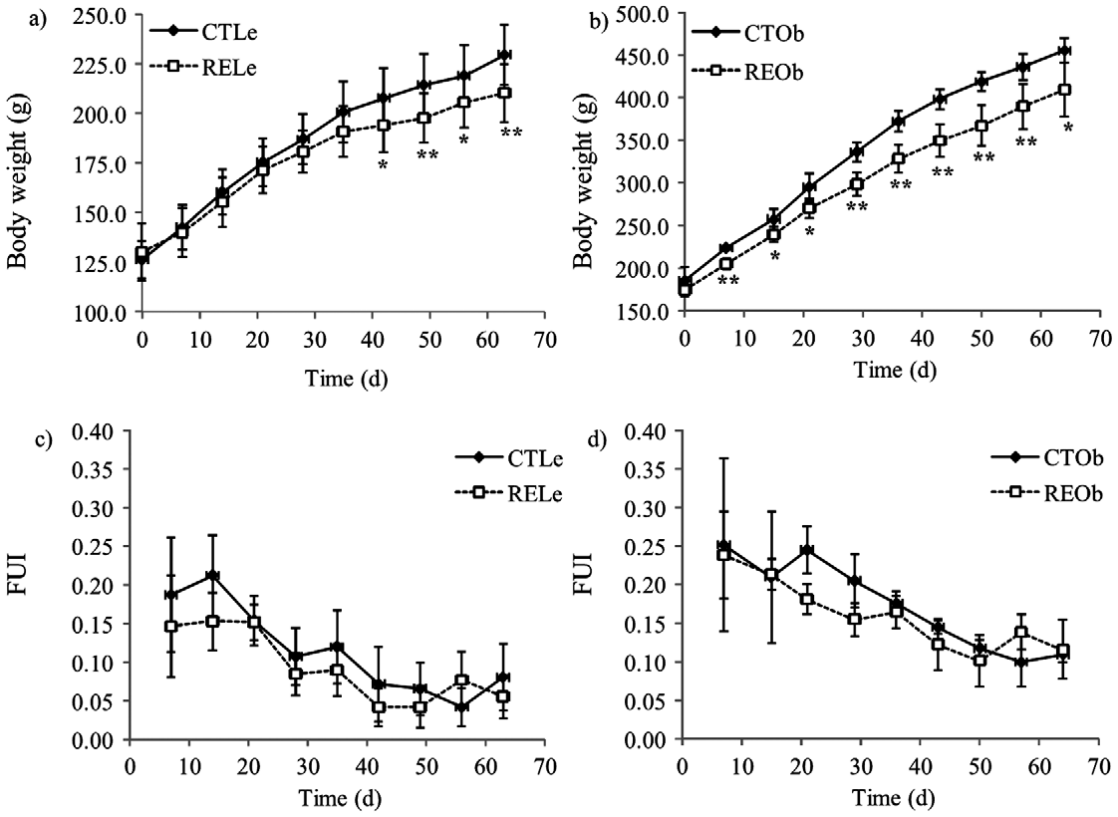
Effects of the consumption of RE on body weight (g) and food utility index (FUI) in Zucker female rats. Lean (Le) and obese (Ob) rats were fed the control diet (CT) or the diet supplemented with 0.05% of RE (RE) for 64 days. Data are presented as the mean value ± SD (n = 7 for lean animals and n = 5 for obese animals). * *P*<0.05, ** *P*<0.01 compared to their respective CT values.

Lean female Zucker rats were markedly smaller than their obese counterparts throughout the experimental period (Figures 3a and 3b). In both groups of animals, supplementation with RE led to a small but significant reduction in body weight which was manifested earlier in the obese animals. Food intake was higher in the obese animals than in their lean counterparts and was kept constant during the supplementation period with no differences observed between control animals (25.09±2.69 and 14.84±2.08 g rat^−1^ day^−1^ for obese and lean rats, respectively) and their RE-supplemented counterparts (23.92±2.29 and 14.44±1.94 g rat^−1^ day^−1^). There were no significant differences either in the consumption of water between the control and treated groups (23.1±5.4 and 23.0±1.7 mL rat^−1^ day^−1^ for obese rats, and 20.1±1.6 and 20.1±1.1 mL rat^−1^ day^−1^ for lean rats, respectively). A small decrease in the weight gain throughout the experimental procedure was reflected in a parallel decrease in the food utility index (body weight gain divided by food consumption) (Figures 3c and 3d) for both groups of animals but no differences were found between control and the RE-supplemented groups. Fecal weight was also monitored during the experiment and was found to be significantly higher (*P*<0.001) in animals supplemented with RE than in the controls: 6.64±0.37 *vs.* 5.42±0.60 g rat^−1^ day^−1^ (fresh weight, f.w.) for obese animals, and 3.64±0.34 *vs.* 2.77±0.20 g rat^−1^ day^−1^ (f.w.) for lean animals, respectively. Rats in the RE supplemented groups had stools with a softer consistency and a lighter color.

### RE rich in CA decreased serum levels of triglycerides and cholesterol in lean Zucker rats but did not affect hepatic lipids

**Figure 4:**
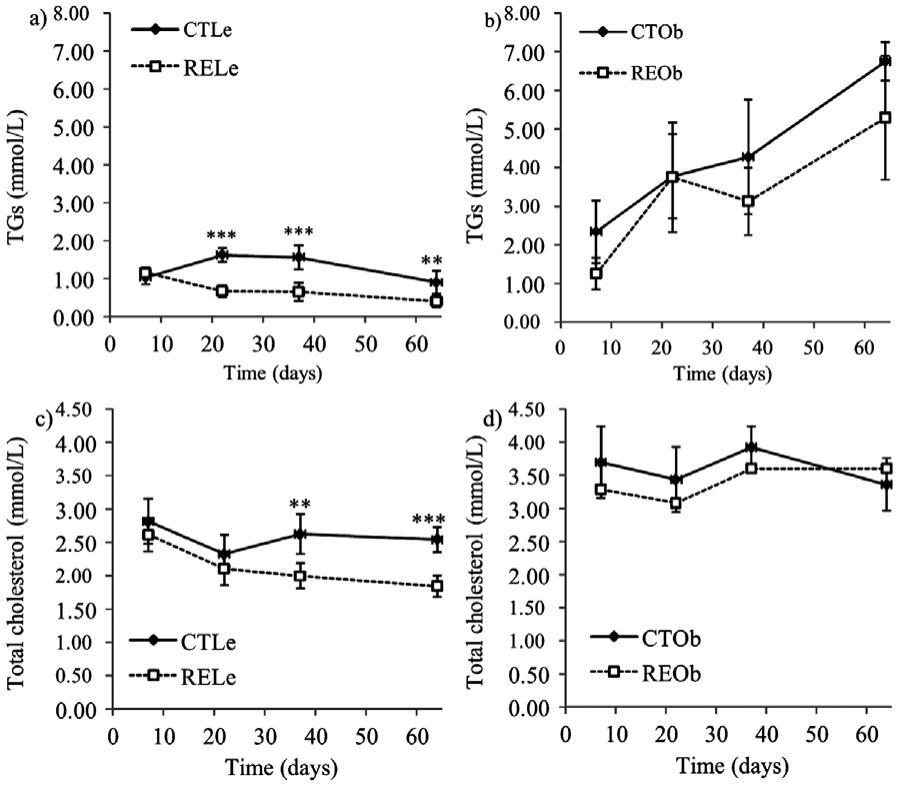
Effects of the consumption of RE on plasma levels of TGs and total cholesterol (mmol/L) in Zucker female rats. Lean (Le) and obese (Ob) rats fed the control diet (CT) or the diet supplemented with RE (RE). Data are presented as the mean value ± SD (n = 7 for lean animals and n = 5 for obese animals). ** *P*<0.01, *** *P*<0.001 compared to their respective CT values.

A complete list with all the plasma biochemistry results is shown in Table S2. A summary with the most significant changes is presented in Figure 4. Lean Zucker female rats had lower levels of TGs (Figure 4a and 4b), total cholesterol (Figures 4c and 4d), LDL-c and HDL-c than their obese counterparts and TGs levels were dramatically increased in the obese animals after the first week of the study (Figure 4b). Supplementation with RE significantly reduced the levels of serum TGs (∼57%) and total cholesterol (∼25%) in the lean animals (Figures 4a and 4c). Although the levels of these two parameters were also slightly lower in the obese RE-supplemented group, differences were neither significant nor consistent throughout the experimental period (Figures 4b and 4d). A significant reduction of the levels of LDL-c and HDL-c was also observed in lean rats consuming RE at the end of the experimental supplementation (64 days). Plasma glucose levels were in the normal range in all the animal groups and were only found to be slightly (but not significantly) increased in the obese rats at the end of the experimental procedure (Table S2). No significant differences were found between the control and the RE-supplemented animals indicating that the RE did not affect the glucose homeostasis during the experimental procedure. Notably, the consumption of the RE enriched in carnosic acid resulted in a significant reduction in plasma insulin levels in the lean rats (0.47±0.27 *vs.* 0.12±0.04 µg/L for control and treated animals, respectively, *P*<0.01) whereas no significant differences were detected between the control and treated obese rats (3.64±1.36 *vs.* 4.89±2.24 µg/L, respectively).

To examine whether supplementation with RE had any effect on the levels of lipids in the liver, we first measured neutral lipid content in liver tissue using oil red O staining. At the end of the experimental procedure, lipid droplets were markedly higher in the obese animals than in the lean ones (data not shown). Following exposure to the RE, no significant differences were observed between control and treated rats. We further measured the levels of TGs and total cholesterol in the liver and found that consumption of the RE did not cause any change in the liver content of TGs (23.4±7.7 mg/g *vs.* 24.8±8.2 mg/g for control and supplemented obese animals, respectively; 16.9±4.3 mg/g *vs.* 17.3±7.5 mg/g for control and RE supplemented lean animals, respectively) or in the content of total cholesterol (1.7±0.1 mg/g *vs.* 1.7±0.2 mg/g for control and RE supplemented obese animals, respectively; 2.0±0.1 mg/g *vs.* 2.0±0.5 mg/g for control and supplemented lean animals, respectively).

### RE rich in CA delivered a high content of intact CA and carnosol to the stomach and significantly inhibited gastric lipase activity

Table 2 shows the final organ and gut content weight in the lean and obese animals as well as CA and carnosol content (µg/g. f.w.) in these organs. Along the gastrointestinal tract the levels of carnosol were always higher than those of CA. The greatest content of carnosol and CA was found in the stomach with total values (CA+carnosol) being higher in the obese animals than in the lean ones: 91.2 µ/g *vs.* 68.8 µ/g. The levels of these two compounds were much lower in the duodenum and in the small intestine where CA was not detected. In the liver, slightly higher levels of CA were found compared to carnosol, whereas neither compound could be detected in the pancreas. In addition, substantial quantities of methyl carnosate were found in all the gut content samples and in the liver.

**Table 2:** Final body weight, organ and gut content weight for lean (Le) and obese (Ob) female Zucker rats fed the control diet (CT) or the diet supplemented with RE enriched in CA (40%).

		CTLe	RELe	CTOb	REOb
Stomach content	Weight (g)^a^	2.77±0.83^b^	2.47±0.77	4.14±1.05	6.28±1.95
	CA (µg/g)	N.D.	24.79±17.67	N.D.	28.09±15.45
	Carnosol (µg/g)	N.D.	43.97±11.22	N.D.	63.09±12.08
	Methyl carnosate (µg/g)	N.D.	11.45±1.59	N.D.	17.26±3.66
Duodenum content	Weight (g)	0.44±0.09	0.48±0.11	0.51±0.08	0.54±0.15
	CA (µg/g)	N.D.	3.32±8.79	N.D.	N.D.
	Carnosol (µg/g)	N.D.	5.54±12.55	N.D.	8.38±5.68
	Methyl carnosate (µg/g)	N.D.	28.03±16.48	N.D.	21.46±11.71
Small intestine^c^ content	Weight (g)	2.44±0.66	2.70±0.25	3.10±0.43	3.30±0.86
	CA (µg/g)	N.D.	N.D.	N.D.	N.D.
	Carnosol (µg/g)	N.D.	12.78±2.92	N.D.	14.55±2.52
	Methyl carnosate (µg/g)	N.D.	12.27±1.29	N.D.	12.88±0.58
Liver	Weight (g)	8.53±0.71	9.21±0.77	14.80±1.70	17.02±1.21
	CA (µg/g)	N.D.	4.15±1.04	N.D.	3.42±1.83
	Carnosol (µg/g)	N.D.	1.63±0.35	N.D.	2.57±1.10
	Methyl carnosate (µg/g)	N.D.	21.84±3.71	N.D.	29.14±9.52
Pancreas	Weight (g)	0.66±0.09	0.68±0.09	0.64±0.11	0.58±0.20
	CA (µg/g)	N.D.	N.D.	N.D.	N.D.
	Carnosol (µg/g)	N.D.	N.D.	N.D.	N.D.
	Methyl carnosate (µg/g)	N.D.	N.D.	N.D.	N.D.

The content of CA, carnosol and methyl carnosate detected in these samples is also indicated.

a : Organs and gut content were collected and weighed at the end of the experimental procedure.

b : Values are the mean ± SD (n = 7 for lean animals and n = 5 for obese animals) and are expressed as g of fresh weight (f.w.);

c : jejunum+ileum; N.D.: Not detected.

**Figure 5:**
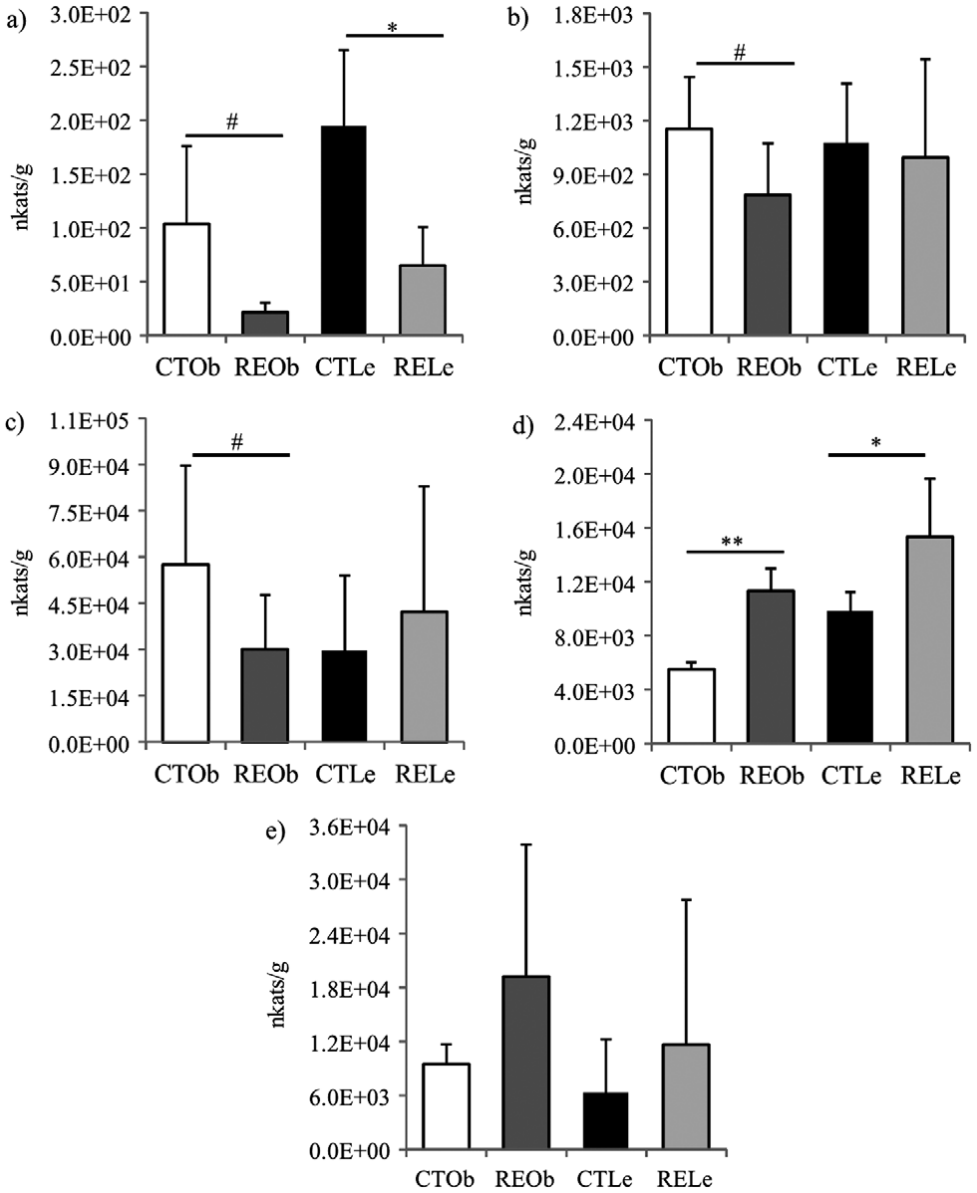
Effects of the consumption of RE on lipase activity in Zucker female rats. Lean (Le) and obese (Ob) rats fed the control diet (CT) or the diet supplemented with RE (RE). Activity (expressed in nkats/g) was measured as the hydrolysis of PNPB in a) stomach content, b) duodenum content, c) small intestine (jejunum+ileum) content, d) liver and e) pancreas. Data are presented as the mean value ± SD (n = 7 for lean animals and n = 5 for obese animals). ^#^ *P*<0.1, * *P*<0.05 and ** *P*<0.01 compared to their respective CT values.

The effects of consumption of RE on lipase activity were determined by monitoring the activity against PNPB in the gut content and organs from the different animal groups. The results are presented in Figure 5. A significant inhibition of the enzyme activity was detected in the stomach content of the obese and lean rats supplemented with RE (approximately 80% inhibition, *P*<0.1 and 70% inhibition, *P*<0.05, respectively) (Figure 5a). The obese rats also exhibited a smaller activity in the duodenum and small intestine content when supplemented with the RE although differences were not significant (Figures 5b and 5c). In contrast, we found that the enzyme activity was significantly induced in the liver of both the obese and lean rats fed with the diet supplemented with RE (approximately100% increase, *P*<0.01 and 50% increase, *P*<0.05, respectively) (Figure 5d). We also observed an increase in the liver weight (Table 2). Similarly, the pancreas of the RE-supplemented animals also exhibited a slightly higher activity but values did not reach significance (Figure 5e).

We additionally tested the capacity of CA and carnosol to inhibit the activity of porcine pancreatic lipase under the conditions of our assay (Figure S1). We found that both compounds exhibited a similar ability to inhibit the hydrolysis of PNPB in a concentration dependent manner and reached around 65–70% inhibition at the highest concentration tested (18.2 µg/mL).

## Discussion

Long-term studies in animals exhibiting obesity are essential to investigate anti-obesity treatments and, the choice of both the animal model and sex are critical points that need to be considered. In the present study we investigated the effects of the daily consumption of a RE highly enriched in CA (40%) on female Zucker lean and obese rats. Although, current worldwide increases in obesity cannot be accounted for by changes in genetic background, single gene mutation animal models such as the *fa/fa* leptin signaling-deficient Zucker rat have been widely used as a model of obesity and metabolic syndrome and provide a very good model for insights into the mechanisms underlying the metabolic deregulation associated to obesity [16]. In contrast to the most generally accepted dietary-induced obesity models [17], the Zucker obese phenotype becomes visible within a few weeks of birth [18] and allows for more rapid evaluation of the effects of anti-obesity compounds. Since males and females exhibit different lipid metabolism [19], body fat distribution and percentage, as well as different levels and sensitivity to key metabolic hormones such as leptin or insulin [17], the choice of sex is also an important issue in studies looking at the effects of anti-obesity drugs. Sexual dimorphism in lipid metabolism is the result of hormone action (estrogens and estrogen receptors, ER) [20] and of other complex sex-specific modulators that remain to be elucidated [21]. After reviewing the literature for studies looking at the anti-obesity effects of carnosic acid or rosemary extract [6]–[9], [22] (see Table 3 of the manuscript) we noticed that the lipid-lowering and body weight-reducing effects of these products had only been tested and reported in male mice and male rats and thus, we carried out our studies in female rats to determine whether these effects were also manifested in females. Of note, carnosol, found at significant levels in the RE and in the gastrointestinal tract and liver after the consumption of the RE, has been reported to interact with ER-alpha and functions as an antagonist [23]. It is then plausible that this molecule may interact with ER in vivo and cause different modulatory effects in female and males.

**Table 3:** Comparative results of some of the reported effects of the consumption of RE or CA on body weight and lipid changes.

Animal model	Tested material	Dose and duration	Body weight	Serum TGs	Serum cholesterol	Liver TGs	Reference
High fat-fed male mice	CA	20 mg/Kg,14 days	↓∼24%	↓∼30%	N.D.	N.D.	[9]
*Ob/ob* male mice	CA	60 mg/Kg,35 days	↓∼20%	↓∼60%	↓∼24%	↓∼47%	[8]
Male rats	RE	250 mg/Kg, 500 mg/Kg,63 days	No significant effect	No significant effect	No significant effect	N.D.	[21]
High fat -fed male mice	RE	200 mg/Kg (≈5 mg CA/Kg and 11.2 mg CAR/Kg),50 days	↓∼64%	No significant effect	No significant effect	↓∼39%	[6]
High fat -fed male mice	RE	600 mg/Kg (≈120 mg CA/Kg),112 days	↓∼25%	No significant effect	↓∼18%	N.D.	[7]

RE: Rosemary extract; CA: carnosic acid; N.D.: Not determined.

Our results show that the consumption of the RE rich in carnosic acid causes a moderate but significant reduction in body weight (approximately 15%) both in the female obese rats and in their lean littermates without affecting the food intake and corroborates with earlier published results in male mice [6]–[9], [22]. Our study and others [6]–[9] also show that these effects were manifested shortly after the start of supplementation with RE or CA (between 10 and 15 days) and were independent of the dose administered or the animal model studied (either diet-induced obesity or genetic models). In this study, rats were fed a standard chow supplemented with 0.5% of RE and thus the effective administered doses ranged approximately from: i) 584 mg RE/Kg body weight (b.w.) day^−1^ (233 mg CA/Kg b.w. day^−1^) at the start of the study to 348 mg RE/Kg b.w. day^−1^ (138 mg CA/Kg b.w. day^−1^) after 64 days of consumption of the extract for the lean rats and, ii) 700 mg RE/Kg b.w. day^−1^ (280 mg CA/Kg b.w. day^−1^) to 544 mg RE/Kg b.w. day^−1^ (218 mg CA/Kg b.w. day^−1^) for the obese animals. The human equivalent doses (HED) [24] would range approximately between 50 and 100 mg of RE/Kg b.w day^−1^ (equivalent to 20 to 40 mg of CA/Kg b.w. day^−1^). These doses would contain a small amount of carnosol (3 to 6 mg/Kg b.w. day^−1^). These quantities of CA+carnosol are well above those estimated to be attained through the diet, i.e. 0.04–0.11 mg/Kg b.w. day^−1^ [25] and could only be achieved through the consumption of nutraceuticals or supplements rich in these compounds for which toxicity has not been fully evaluated.

Regarding the toxic effect of long-term consumption of high doses of REs or CA plus carnosol, the European Food Safety Authority has established that the non-observed-adverse-effects-level (NOAEL) values are in the range of 180 to 400 mg extract/Kg b.w. day^−1^ (equivalent to ∼20–60 mg of CA+carnosol/Kg b.w. day^−1^) based on 90-days feeding studies in rats [25]. It has also been reported that an acute single dose of 2000 mg of RE/Kg b.w. (containing up to 48% CA and 11% of carnosol) was not toxic to Wistar rats as it did not affect hematological and clinical parameters, body and organ weight, or gross or histological characteristics [26]. In the present study, sub-chronic consumption of the aforementioned doses of RE (containing 40% CA and 6.5% carnosol) for 64 days did not substantially affect any of the hematological parameters tested indicating a low toxicity of the extract. This was further supported by the small changes observed in the levels of the hepatic enzymes. We did observe, however, a small increase in the WBCs, primarily in the lymphocytes. Although an increase in these cells might be considered a sign of inflammatory response to infection, the increased values were still within the normal range [26] and they may also suggest a small immunostimulatory effect like that reported for other plant medicinal extracts [27], [28]. A systemic immunomodulation may be a consequence of a modulatory effect on the gut-associated immune function partially associated with activation and modification of the number of Peyer's patches [29]. In support of a putative RE immunostimulatory effect for a RE enriched in CA, we counted the Peyer's patches in the intestine of the lean and obese rats and found that numbers were slightly higher (although not significantly) in the groups supplemented with RE than in control groups (data not shown).

Another important effect attributed to CA or RE in male animals is the reduction of the levels of TGs and cholesterol as summarized in Table 3. The consumption of CA appears to reduce TGs in plasma and this effect seems to be more noticeable at a higher dose. CA also seems to be able to reduce the levels of TGs in the liver. However, studies evaluating the effects of the consumption of RE containing CA have yielded less consistent results. Discrepancies may be due to variability in the composition of the extract and the doses of the RE used, or to the animal models employed. In our study, body weight was reduced in the lean and obese animals indicating that both phenotypes were responding to treatment with the RE. However, the decrease in the levels of TG and cholesterol was only significant in the lean female rats. These differences may be attributed to the altered lipolytic responsiveness in the obese Zucker rats that can be refractory to nutritional and pharmacological interventions [30]. The obese animals have a well-developed hyperlipidemia with rather high serum lipid levels where it is more difficult to exert a regulatory effect. Recently, it has been suggested that the lack of a functional leptin receptor in the obese *fa/fa* Zucker rats contributes to the impairment of the regulation of fat metabolism and may worsen or slow the capacity of these animals to mobilize lipids [31]. In contrast to the obese animals, the lean Zucker rats are considered normal rats, sensitive to insulin, normotensive and with a normal glucose tolerance and lipid profile [16]. The levels of key metabolic regulators are also different in the lean animals as compared to the obese ones [32]. These differences may account for their different responses to dietary factors, especially those that affect the lipid metabolism and therefore it is not unusual to find differences between the responses of lean and obese Zucker rats after exposure to anti-obesity drugs. Like this, a fermented-milk product caused a significant decrease in body weight, mesenteric fat, mesenteric adipocyte size, and HDL levels in lean rats but not in the obese animals [33] or oral fibrate administration significantly lowered serum lipids in lean animals only [34]. Zucker obese animals are not always resistant to antiobesity therapies and, for example, treatment with oleoylethanolamide decreased body weight more markedly in lean Zucker rats than in their obese counterparts but the TG levels were not affected in the lean animals and were significantly reduced in the obese animals [35], [36]. With regard to the insulin levels, our data are in good agreement with those reported by Sánchez et al. [12] for female lean and obese control rats. These authors also reported a reduction in the levels of plasma insulin in Zucker rats fed a fiber-enriched diet. A link between dietary fiber, lower insulin levels and decreased body weight has been already described [37]. Insulin is also a very important regulator of plasma TG homeostasis by upregulating TG synthesis and suppressing TG hepatic secretion [38] with usually a direct correlation between TGs and insulin levels. Our results show that the observed reduction in the levels of TG in the lean Zucker rats was concomitant with a reduction in the serum insulin levels.

The reduction in body weight and lipid levels observed after the consumption of RE or CA has been suggested to be caused by a reduction in the absorption of dietary fat further supported by an increase in fecal fat excretion [6], [7]. Although we did not measure the fat content in the feces in our study, we did find a significant increase in the fecal mass as well as a paler color of the feces in the RE-supplemented animals consistent with a higher content in fat. Since RE, CA and also carnosol are able to inhibit the activity of pancreatic lipase *in vitro*, the reduction in body weight and lipid levels may be mediated through the inhibition of pancreatic lipase activity in the gastrointestinal tract [6], [7], [9]. To determine if this lipase inhibition may occur in vivo, it is important to evaluate: i) whether these compounds may reach sufficient concentrations in the gastrointestinal tract in their intact molecular form and if so, ii) whether they are able to inhibit the enzyme activity in the gut. *In vitro* studies have shown that under simulated conditions of the gastric digestion (pH = 2.0, 37°C, 1 h) the concentration of CA, carnosol and also of methyl carnosate is diminished. This decrease is even greater under simulated conditions of the intestinal digestion (pH = 7.5, 37°C, 1 h) and some conversion of CA into carnosol has been reported [39]. However, these conditions may differ from those observed *in vivo* where after feeding the pH may be around 3.9 in the stomach and below 6.6 in the intestine of rats [40] and thus, *in vivo* results may be different. In rats, an intra-gastric acute dose of CA (90 mg/Kg) yielded high quantities of CA in the stomach (maximum concentration detected was approximately 300 µg/g) whereas, in the intestine, the quantity of CA was 10-fold lower (30 µg/g). Measurements of carnosol content were not reported in this study [41]. We have quantified the levels of CA, carnosol and methyl carnosate present in the gastrointestinal tract of the Zucker rats supplemented with RE and found the highest levels of CA and carnosol in the stomach. Along the gastrointestinal tract, we also found that the levels of CA were always lower than those of carnosol. These results support some *in vivo* conversion of one compound to another but it may also be due to a faster absorption of CA than of carnosol. Recently, it has been reported that CA can be detected in the plasma of rats 7 min after oral dosage of the compound [42]. Interestingly, of the three compounds detected, the most abundant in the jejunum, ileum, and liver, was the methylated derivative of CA. Although this metabolite may originate partially from the compound already present in the RE, our data suggest an *in vivo* methylation of CA likely due to the action of cathecol-*O*-methyl transferases (COMTs) present in the intestine and liver [43].

Our data also showed that the highest and most significant inhibition of lipase activity (70–80%) was detected in the stomach of the RE-supplemented animals. We have also shown that *in vitro* inhibition of lipase activity by CA and carnosol was around 65–70% for a test compound concentration of ∼18 µg/mL. Based on the quantities of CA and carnosol found in the stomach of the rats and given an estimated stomach volume of 3.5 mL [40], the concentrations of these compounds in the stomach of the Zucker rats may reach values as high as ∼18 µg/mL and ∼50 µg/mL of CA for the lean and obese animals, respectively, whereas carnosol may be even higher, ∼30 µg/mL and ∼100 µg/mL. These concentrations substantiate the pronounced lipase activity inhibition observed in the stomach content. The main lipase activity in the stomach is due to gastric lipase (GL) which is an active and stable lipolytic enzyme that initiates the gastrointestinal digestion of dietary fat [44]. GL continues its activity in the duodenum where it acts synergistically with pancreatic lipases and may be able to release 10–40% of dietary triglyceride acyl chains [2]. Therefore, inhibition of GL may contribute greatly to reduce the absorption of fat. Other dietary phenolic compounds, such as cocoa procyanidins [15], tea polyphenols [45] or hydroxytytrosol [46] have also been reported to inhibit pancreatic lipase activity *in vitro*. We speculate that they may also be able to inhibit gastric lipase and thus, regular consumption of high quantities of phenolic compounds through the diet or dietary supplements may supply sufficient quantities into the stomach, where they remain structurally unmodified [47], and efficiently reduce the digestion and subsequent absorption of fat. Further research looking at the inhibition of enzymatic fat digestion in the stomach by RE and its main phenolic components is warranted.

Orlistat is a well-known pancreatic lipase inhibitor which may cause weight loss of up to 10% [48]. Low doses of orlistat (10–20 mg/Kg/day) can also decrease serum lipid levels and modulate food intake although results show some variability [49]–[52]. In our study, the RE rich in carnosic acid, at oral doses between 350 and 700 mg extract/Kg/day (140 to 280 mg carnosic/Kg/day), caused a significant reduction in body weight (∼15%) without affecting food intake. A significant reduction in TG and cholesterol was also shown in the lean animals. These results indicate a good efficiency of the extract as compared to that of orlistat. Although the doses of RE were higher than those of the drug, we need to bear in mind that the extract include many other active or inactive components and, consequently, the inhibitory potency of the extract may result diluted. Like this, in vitro testing of the lipase activity inhibition clearly shows that orlistat is more potent than a RE containing 20% carnosic acid [7]. However, in an acute test carried out in oil-loaded male mice, the same oral doses of pure carnosic acid and orlistat (5 to 20 mg per Kg) caused a similar reduction in serum TG in a maximum period of 6 h [9] indicating a similar efficiency between orlistat and carnosic acid.

In addition, in this study we also found that the livers of the animals supplemented with RE had a higher weight and a significant induction of the enzymatic activity. These results are in agreement with previous data reporting liver enlargement and enzyme induction as a response to exposure to rosemary extracts [53], [54]. However, the levels of CA and carnosol detected in the liver were much lower than those in the gut which suggests that other derived metabolites may be responsible for the effects in this organ. Further ongoing research is being carried out at our lab to i) identify other CA and carnosol metabolites present in the liver and ii) to determine gene and protein changes induced in the liver of the supplemented animals that may contribute to explain the modulation of the plasma lipid levels caused by RE consumption as well as the differences between lean and obese animals. In conclusion, our results show a significant inhibition of gastric lipase in the stomach of Zucker rats consuming RE enriched in CA which may cause a moderate reduction of fat absorption consistent with the observed reduction in weight gain and triglycerides and cholesterol levels. Our data suggest that long-term consumption of rosemary extracts rich in CA may be beneficial for maintaining a normal lipid profile and a lower weight.

## Supporting Information

Figure S1 ***In vitro*** **inhibition of pancreatic lipase by carnosic acid and carnosol.** Carnosic acid or carnosol (5, 10 and 20 µg) were mixed with 20 µL of porcine pancreatic lipase (Type II, 100–400 units/mg protein; 1 mg/mL) in 0.1 M Tris-HCl buffer (pH 8) and 25 µL *p*-nitrophenylbutyrate (PNPB; 2 mM) were added to start the reaction (final volume, 1100 µL). Following incubation at 37°C for 5 min, absorbance was read at 400 nm. Values are presented as % of inhibition respect to control values in the absence of test compound. Measurements were done in triplicate and results are presented as mean values ± SD.
(DOCX)

Table S1 **Hematological parameters in female Zucker rats.**
(DOCX)

Table S2 **Biochemical parameters in female Zucker rats.**
(DOCX)
