# Supplementary material for: Inhibition of Gastric Lipase as a Mechanism for Body Weight and Plasma Lipids Reduction in Zucker Rats Fed a Rosemary Extract Rich in Carnosic Acid
Source: PLoS One. 2012 Jun 22;7(6):e39773. doi: 10.1371/journal.pone.0039773 (PMC3382157; doi:10.1371/journal.pone.0039773)
Supplement: Table S1 — Hematological parameters in female Zucker rats. (DOCX) [file pone.0039773.s002.docx]

**Table S1.** Hematological parameters in female Zucker rats lean (Le) and obese (Ob) after 64 days of consumption of a standard chow (CT) or the standard chow supplemented with RE (RE) containing 40% carnosic acid.

| Parameter | CTLe | RELe | CTOb | REOb |
| --- | --- | --- | --- | --- |
| WBC (×10^3^/µL) | 5.89 ± 1.33^a^ | 6.84 ± 1.19 | 5.30 ± 0.22 | 6.84 ± 1.51 |
| Lymphocytes (×10^3^/µL) | 3.67 ± 0.66 | 4.58 ± 0.74* | 3.05 ± 0.43 | 4.23 ± 1.20* |
| Lymphocytes (%) | 63.39 ± 8.03 | 67.26 ± 5.59 | 57.38 ± 6.07 | 55.48 ± 11.03 |
| MID | 0.25 ± 0.13 | 0.26 ± 0.08 | 0.17 ± 0.08 | 0.36 ± 0.06* |
| Granulocytes (×10^3^/µL) | 1.96 ± 0.86 | 2.00 ± 0.57 | 2.08 ± 0.33 | 2.25 ± 0.55 |
| Granulocytes (%) | 32.24 ± 8.15 | 28.89 ± 4.87 | 39.30 ± 7.16 | 33.22 ± 6.23 |
| RBC (×10^6^/µL) | 8.09 ± 0.23 | 8.29 ± 1.63 | 7.92 ± 0.26 | 7.75 ± 0.32 |
| Hemoglobin (g/dL) | 15.57 ± 0.87 | 15.71 ± 2.39 | 15.22 ± 0.35 | 15.40 ± 2.25 |
| Hematocrit (%) | 43.83 ± 2.05 | 45.01 ± 7.53 | 42.51 ± 2.46 | 41.28 ± 1.64 |
| MCV (fL) | 54.14 ± 2.54 | 54.57 ± 3.64 | 53.60 ± 1.95 | 53.40 ± 0.55 |
| MCH (pg) | 19.24 ± 0.79 | 19.07 ± 0.94 | 19.24 ± 0.41 | 19.82 ± 2.01 |
| MCHC (g/dL) | 35.56 ± 1.89 | 34.99 ± 1.12 | 35.84 ± 1.79 | 37.20 ± 3.93 |
| RDW (%) | 16.79 ± 1.23 | 16.74 ± 0.76 | 18.66 ± 0.72 | 17.56 ± 0.58 |
| Platelets (×10^3^/µL) | 616.57 ± 54.02 | 569.86 ±331.95 | 642.00 ± 65.10 | 746.20 ± 96.96 |
| Plateletcrit (%) | 0.37 ± 0.02 | 0.35 ± 0.24 | 0.39 ± 0.05 | 0.46 ± 0.05 |
| MPV (fL) | 6.01 ± 0.21 | 6.00 ± 0.61 | 6.10 ± 0.21 | 6.22 ± 0.29 |
| PDW (%) | 32.6 ± 0.33 | 31.66 ± 1.76 | 32.84 ± 0.46 | 33.60 ± 1.20 |

^a^ : Data are presented as the mean value ± SD (n=7 for lean animals and n=5 for obese animals). WBC, white blood cell count; MID, mid-cell fraction; RBC, red blood cell count; MCV, mean corpuscular volume; MCH, mean corpuscular hemoglobin; MCHC, mean corpuscular hemoglobin concentration; RDW, red blood cell distribution width; MPV, mean platelet volume; PDW, platelet distribution width. *: *P* < 0.05 compared to their respective CT value.
