# Supplementary material for: Inhibition of Gastric Lipase as a Mechanism for Body Weight and Plasma Lipids Reduction in Zucker Rats Fed a Rosemary Extract Rich in Carnosic Acid
Source: PLoS One. 2012 Jun 22;7(6):e39773. doi: 10.1371/journal.pone.0039773 (PMC3382157; doi:10.1371/journal.pone.0039773)
Supplement: Table S2 — Biochemical parameters in female Zucker rats. (DOCX) [file pone.0039773.s003.docx]

**Table S2.** Plasma levels of triglycerides (TGs), total cholesterol (T-Chol), LDL cholesterol (LDL-c), HDL cholesterol (HDL-c), glucose, alkaline phosphatase (ALP), aspartate transaminase (AST) and alanine transaminase (ALT) in female Zucker rats lean (Le) and obese (Ob) after consumption for 64 days of a standard chow (CT) or a standard chow supplemented with RE containing 40% carnosic acid.

| TGs (mmol/L)^a^ | | | | |
| --- | --- | --- | --- | --- |
|  | CTLe | RELe | CTOb | REOb |
| 7 days | 1.02 ± 0.18 | 1.15 ± 0.15 | 2.34 ± 0.82 | 1.24 ± 0.41 |
| 22 days | 1.62 ± 0.19 | 0.66 ± 0.16*** | 3.77 ± 1.09 | 3.75 ± 1.42 |
| 37 days | 1.56 ± 0.32 | 0.65 ± 0.24*** | 4.27 ± 1.49 | 3.12 ± 0.87 |
| 64 days | 0.90 ± 0.30 | 0.40 ± 0.18** | 6.75 ± 0.50 | 5.28 ± 1.61 |
| T-Chol (mmol/L) | | | | |
| 7 days | 2.81 ± 0.34 | 2.62 ± 0.25 | 3.70 ± 0.54 | 3.29 ± 0.37 |
| 22 days | 2.32 ± 0.29 | 2.11 ± 0.26 | 3.44 ± 0.49 | 3.08 ± 0.87 |
| 37 days | 2.62 ± 0.30 | 2.00 ± 0.20** | 3.92 ± 0.32 | 3.60 ± 0.60 |
| 64 days | 2.54 ± 0.19 | 1.84 ± 0.16** | 3.36 ± 0.40 | 3.61 ± 0.19 |
| LDL-c (mmol/L) | | | | |
| 7 days | 0.53 ± 0.09 | 0.51 ± 0.10 | 1.20 ± 0.51 | 0.47 ± 0.13 |
| 22 days | 0.51 ± 0.02 | 0.51 ± 0.08 | 1.28 ± 0.61 | 0.98 ± 0.42 |
| 37 days | 0.44 ± 0.11 | 0.36 ± 0.02 | 0.89 ± 0.01 | 0.71 ± 0.07 |
| 64 days | 0.80 ± 0.09 | 0.58 ± 0.08** | 1.00 ± 0.26 | 1.21 ± 0.13 |
| HDL-c (mmol/L) |  |  |  |  |
| 7 days | 1.67 ± 0.14 | 1.57 ± 0.15 | 2.22 ± 0.20 | 2.36 ± 0.45 |
| 22 days | 1.33 ± 0.06 | 1.20 ± 0.14 | 1.69 ± 0.16 | 1.69 ± 0.10 |
| 37 days | 1.40 ± 0.17 | 1.23 ± 0.10* | 1.87 ± 0.08 | 1.94 ± 0.15 |
| 64 days | 1.34 ± 0.11 | 1.12 ± 0.08** | 1.56 ± 0.15 | 1.80 ± 0.07* |
| Glucose (mmol/L) | | | | |
|  | CTLe | RELe | CTOb | REOb |
| 7 days | 8.01 ± 0.90 | 7.61 ± 0.41 | 6.61 ± 0.72 | 5.91 ± 0.54 |
| 22 days | 7.66 ± 0.48 | 7.17 ± 0.67 | 7.47 ± 0.39 | 7.00 ± 0.24 |
| 37 days | 8.22 ± 1.17 | 7.70 ± 1.05 | 5.70 ± 0.22 | 7.03 ± 0.83 |
| 64 days | 8.86 ± 1.94 | 9.21 ± 2.77 | 12.28 ± 2.52 | 10.78 ± 3.01 |
| ALP (U/L) |  |  |  |  |
| 7 days | 336.87 ± 116.72 | 397.94 ± 58.66 | 432.32 ± 63.87 | 406.05 ± 32.34 |
| 22 days | 240.68 ± 63.20 | 602.20 ± 271.20* | 269.92 ± 39.85 | 425.50 ± 149.19 |
| 37 days | 183.89 ± 39.40 | 341.90 ± 117.77* | 258.38 ± 31.14 | 341.88 ± 92.44 |
| 64 days | 170.50 ± 17.98 | 335.89 ± 125.85* | 186.92 ± 28.23 | 218.78 ± 57.88 |
| AST (U/L) |  |  |  |  |
| 7 days | 119.30 ± 27.00 | 123.60 ± 35.20 | 136.00 ± 27.90 | 145.10 ± 44.60 |
| 22 days | 108.60 ± 35.80 | 110.70 ± 27.80 | 117.30 ± 30.20 | 106.20 ± 31.00 |
| 37 days | 132.80 ± 59.90 | 105.20 ± 44.30 | 149.20 ± 58.00 | 99.00 ± 28.50 |
| 64 days | 90.60 ± 28.70 | 76.50 ± 30.40 | 95.60 ± 13.60 | 95.40 ± 15.40 |
| ALT (U/L) |  |  |  |  |
| 7 days | 59.70 ± 8.60 | 66.30 ± 21.10 | 95.00 ± 23.30 | 74.00 ± 25.40* |
| 22 days | 58.20 ± 7.20 | 55.40 ± 10.70 | 75.50 ± 3.10 | 68.40 ± 7.30 |
| 37 days | 64.30 ± 14.20 | 48.00 ± 4.80 | 66.30 ± 9.10 | 58.30 ± 9.70 |
| 64 days | 39.20 ± 5.00 | 41.60 ± 6.30 | 55.80 ± 6.50 | 46.90 ± 5.30* |

^a^: Data are presented as the mean value ± SD (n=7 for lean animals and n=5 for obese animals). *: *P* < 0.05, ** *P* < 0.01, *** *P* < 0.001, compared to their respective CT value.
