# Supplementary material for: Inhibition of Gastric Lipase as a Mechanism for Body Weight and Plasma Lipids Reduction in Zucker Rats Fed a Rosemary Extract Rich in Carnosic Acid
Source: PLoS One. 2012 Jun 22;7(6):e39773. doi: 10.1371/journal.pone.0039773 (PMC3382157; doi:10.1371/journal.pone.0039773)
Supplement: Supplementary file 3 [file pone.0039773.s001.docx]

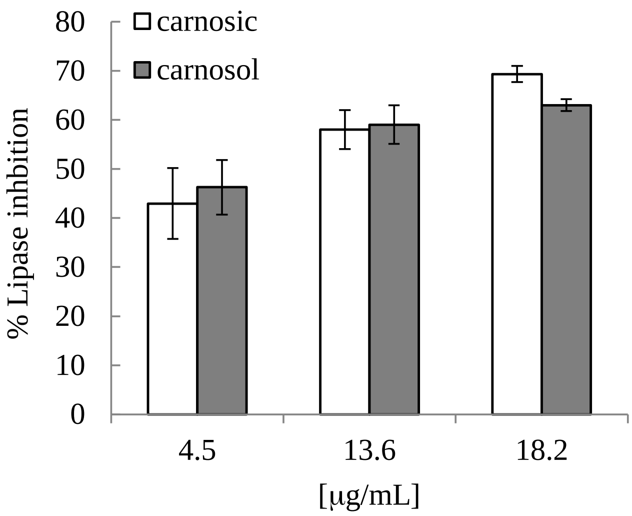

carnosic
carnosol
% Lipase inhbition
80
70
60
50
40
30
20
10
0
4.5
13.6
18.2
[μg/mL]
